# Single nucleotide polymorphisms for feed efficiency and performance in crossbred beef cattle

**DOI:** 10.1186/1471-2156-15-14

**Published:** 2014-01-30

**Authors:** Mohammed K Abo-Ismail, Gordon Vander Voort, James J Squires, Kendall C Swanson, Ira B Mandell, Xiaoping Liao, Paul Stothard, Stephen Moore, Graham Plastow, Stephen P Miller

**Affiliations:** 1 Centre for Genetic Improvement of Livestock, Department of Animal and Poultry Science, University of Guelph, Guelph, Ontario N1G 2W0, Canada; 2 Department of Animal and Poultry Science, Damanhour University, Damanhour, Egypt; 3 Animal Sciences Department, North Dakota State University, Fargo, ND, USA; 4 Livestock Gentec, University of Alberta, Edmonton, AB, Canada; 5 Queensland Alliance Agr & Food Innovation, University of Queensland, St Lucia, Qld 4072, Australia; 6 Animal and Poultry Science Department, Ontario Agriculture College, University of Guelph, 50 Stone Road, Guelph, ON N1G 2W1, Canada

**Keywords:** Candidate genes, Single nucleotide polymorphism, Feed efficiency, Carcass traits

## Abstract

**Background:**

This study was conducted to: (1) identify new SNPs for residual feed intake (RFI) and performance traits within candidate genes identified in a genome wide association study (GWAS); (2) estimate the proportion of variation in RFI explained by the detected SNPs; (3) estimate the effects of detected SNPs on carcass traits to avoid undesirable correlated effects on these economically important traits when selecting for feed efficiency; and (4) map the genes to biological mechanisms and pathways. A total number of 339 SNPs corresponding to 180 genes were tested for association with phenotypes using a single locus regression (SLRM) and genotypic model on 726 and 990 crossbred animals for feed efficiency and carcass traits, respectively.

**Results:**

Strong evidence of associations for RFI were located on chromosomes 8, 15, 16, 18, 19, 21, and 28. The strongest association with RFI (P = 0.0017) was found with a newly discovered SNP located on BTA 8 within the ELP3 gene. SNPs rs41820824 and rs41821600 on BTA 16 within the gene HMCN1 were strongly associated with RFI (P = 0.0064 and P = 0.0033, respectively). A SNP located on BTA 18 within the ZNF423 gene provided strong evidence for association with RFI (P = 0.0028). Genomic estimated breeding values (GEBV) from 98 significant SNPs were moderately correlated (0.47) to the estimated breeding values (EBVs) from a mixed animal model. The significant (P < 0.05) SNPs (98) explained 26% of the genetic variance for RFI. *In silico* functional analysis for the genes suggested 35 and 39 biological processes and pathways, respectively for feed efficiency traits.

**Conclusions:**

This study identified several positional and functional candidate genes involved in important biological mechanisms associated with feed efficiency and performance. Significant SNPs should be validated in other populations to establish their potential utilization in genetic improvement programs.

## Background

As feed costs are a major factor influencing the profitability of beef cattle production, there are many endeavors to reduce these costs. Improving feed efficiency can be achieved by novel feeding strategies and genetic improvement technologies. Although residual feed intake (RFI) has emerged as one of the important feed efficiency traits for beef cattle [1], there are limitations with RFI for direct selection to improve feed efficiency industry-wide. These limitations are the expense and difficulty of recording an animal’s daily feed intake. Genomic approaches offer opportunities to select cattle that are more efficient, as once the relationships between genetic markers and feed efficiency are determined, this prediction can be applied to animals that are genotyped, but are not phenotyped with costly feed intake measurements [2].

Since 2000, advances in high-throughput genotyping and sequencing techniques have resulted in high density SNP chips, such as the Illumina BovineSNP50 BeadChip [3] being available. The use of the Bovine SNP50 in dairy cattle has increased the accuracy for predicting the genetic value of animals [4]. In beef cattle, the use of such developments will benefit most traits such as feed efficiency and carcass traits which are difficult to measure or require the animals to be slaughtered for recording their phenotypes [5]. Several genome wide association studies (GWAS) indicated that many genes affect feed efficiency traits and that the majority of these effects are small [6-11]. These studies reported many SNPs conferring genetic variation in feed efficiency. Nonetheless, although many SNPs were studied, the genetic architecture of feed efficiency was not completely explained.

Results from fine-mapping by Abo-Ismail et al. ([12]) suggested a list of candidate genes for further investigation to identify the causal mutations for feed efficiency within these genes [12]. Discovery of the causal mutations within these genes could help explain the genetic architecture of feed efficiency. Furthermore, this approach could provide a panel of the most informative SNPs that could be used to predict feed efficiency accurately and affordably for producers. Therefore, the objectives of this study were to: (1) identify new SNPs for RFI and performance traits within candidate genes identified in previous GWAS studies; (2) estimate the proportion of variation in feed efficiency traits explained by the detected SNPs; (3) estimate the effect of detected SNPs on carcass traits to avoid undesirable correlated effects when selecting for feed efficiency; and (4) map the corresponding genes to a biological process and pathway to understand the biological meaning behind the detected associations. In this way it was hoped to identify causal mutations or to identify markers in strong linkage disequilibrium with such mutations.

## Methods

### Animals and phenotypic data

The study was approved from The University of Guelph Animal Care Committee based on the recommendations outlined in the Canadian Council on Animal Care (1993) guidelines.

#### ***Feed efficiency traits***

Average daily dry matter intake (DMI), average daily gain (ADG), midpoint metabolic weight (MMWT), RFI and feed conversion ratio (FCR) phenotypes were measured on 726 crossbred beef cattle, heifers (38), steers (387), and bulls (301) at the University of Guelph’s Elora Beef Research Center (EBRC). Average breed compositions were formed by Angus (45.9%), Simmental (20.7%), Piedmontese (5%), Gelbvieh (4.2%), Charolais (2%) and Limousin (1.4%). Animals primarily originated from one of two University of Guelph herds (EBRC and NLARS), the Agriculture and Agri-Food Canada Kapuskasing Research Centre (KAP) or were purchased from producers in Ontario, Canada. Calves were weaned at approximately 200 days of age, and were involved in various post-weaning trials at the EBRC with different nutrition treatments. The body weights of the animals were recorded a number of times over the trials with most trials recording weights at least every four weeks.

The ADG for individual animals was calculated as a linear regression coefficient of their live weights on the actual days of measurement using the nlme package from R software [13]. The MMBW was calculated as the midpoint body weight (kg) to the power 0.75. The DMI was calculated for each animal as total DMI divided by number of days for the test period. The RFI was calculated from the difference between the average of the animal’s actual daily DMI and its expected daily DMI [14]. Expected DMI was determined through the regression coefficients estimated from the data through a multiple phenotypic regression model as follows:

(1) $$y_{\mathrm{ijk}}=µ+\beta_{1}\left({\mathrm{ADG}}_{k}\right)+\beta_{2}\left({\mathrm{MWT}}_{k}\right)+{\mathrm{Sex}}_{i}+{\mathrm{TTY}}_{j}+e_{\mathrm{ijk}}$$

Where, y_ijk_ is the total DMI for animal k during the feeding period, μ is the overall mean, β1 is the regression coefficient of the linear regression on ADG as determined through a linear regression of weights on days on trial as described, β2 is the regression coefficient of the linear regression on MMWT, sex_i_ is the effect of i^th^ sex, TTY_j_ is the effect of j^th^ treatment × trial × year (42 levels) and e_ijk_ is the residual random effect associated with the animal k and is the resulting RFI used in further analyses.

#### ***Carcass and meat quality traits***

The association analysis of carcass and meat quality traits was carried out on 693-990 (depending on the trait) crossbred animals, including heifers (n = 33), steers (n = 705), and bulls (n = 252). In total 698 of these animals have RFI measures. All cattle were slaughtered at the University of Guelph Meat Science Laboratory Abattoir. Hot carcass weight (HCW) was measured just before the carcass was placed in the cooler. Meat Laboratory staff assessed the *longissimus* muscle interface (i.e. muscle surface) between the 12^th^ and 13^th^ ribs to obtain the following carcass measurements: subcutaneous fat depths between the 1^st^ and 2^nd^, 2^nd^ and 3^rd^, and 3^rd^ and 4^th^ quadrants of *longissimus* muscle (recorded as F1, F2 and F3, respectively), the grade fat (GRF), the minimum measurement of subcutaneous fat depth within the 4^th^ quadrants of *longissimus* muscle and *longissimus* muscle area, measured using an electronic planimeter (MOP-3; Carl Zeiss Inc., Thornwood, NY) after acetate tracing (Bergen *et al.*[15]). Canadian Beef Grading Agency formulae (http://www.beefgradingagency.ca/) were used to determine lean yield (LY), an estimate of the percentage of the carcass that is red meat. Marbling was assessed to determine the average amount, size and distribution of fat particles or deposits within *longissimus* muscle andwas scored as ≤3.0 = devoid; 3.1 to 4.0 = traces; 4.1 to 5.9 = slight; 6.0 to 7.0 = small to moderate; and ≥7.0 = slightly abundant to abundant. Rib dissection traits were also measured using a 4-6 rib section depending on the trial and year (physical separation of ribs 8-12 or 6-12, respectively). This procedure determines the amount of lean meat and bone, and a quantitative and qualitative assessment of fat depots (body, subcutaneous and intermuscular) within the rib to evaluate carcass composition. A complete description of carcass measurements was discussed by [15].

### SNP discovery, DNA isolation and genotyping

Messenger RNA from seven tissue types (adipose, muscle, hypothalamus, duodenum, liver, lung and kidney) was extracted using TRIzol (Invitrogen). The tissue samples were collected from beef cattle at the Lacombe Research Centre in Alberta (Canada). RNA from 7 to 14 animals was pooled for each tissue before sequencing. Sequencing libraries were constructed from each RNA pool according to a standard protocol (mRNA Sequencing Sample Preparation Guide, Illumina, USA). Sequencing was performed on the Illumina Genome Analyzer II following the manufacturer’s recommendations. The resulting reads (more than 140 M) were mapped to transcript sequences from the reference bovine genome assembly (Btau4.0) [16] using maq 0.6.6 [17]. More than 1.2 million SNPs were detected by comparing the aligned reads to the reference transcripts. From this list a subset of 300 SNPs from 215 candidate genes was selected based on SNP functional consequences assigned by NGS-SNP [18]. An additional 158 coding SNPs were chosen from publicly available SNPs within the same candidate genes (Additional file 1). These genes were selected based on their proximity (on average distance 116,963 base pair) to significant SNPs identified in a previous study [12].

Tissue or blood samples were prepared and sent to Laboratory Services, University of Guelph, for genomic DNA extraction. Then, prepared DNA samples were sent to GeneSeek, Inc. for genotyping using a commercial platform for high-throughput SNP genotyping. In total, 1,032 animals, as assessed by the numerator relationship matrix using CFC, born subsequent to the animals used in the GWAS population [12] were genotyped for 458 SNP. The 300 SNPs identified through this work that were verified through genotyping have been submitted to dbSNP under the handle name “UALG”.

**Figure 1:**
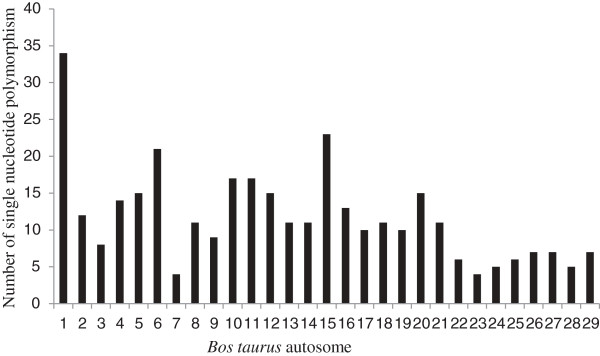
The distribution of 339 genotyped SNP across 29 chromosomes of the bovine genome.

Quality control (QC) was done using the GenABEL package [19] in R software. Animals (n = 14) and individual SNPs (n = 5) with a low call-rate (<90%) were excluded from the analysis. Mean Identical By State (IBS) was 0.783 ± 0.0327. Animals (n = 1) with high estimation of IBS (≥0.95) were excluded. SNPs (n = 114) with a minor allele frequency (MAF) (< 1%) were excluded from the analysis of feed efficiency traits. Mean autosomal heterozygosity (HET) was 0.27 ± 0.036; animals (n = 6) with high HET (≥0.446) were excluded. Three hundred thirty nine SNPs and 727 animals passed all QC criteria where these SNPs were mapped to 180 corresponding genes (83,58, 24, 9, 4, and 2 genes including only 1, 2, 3, 4, 5 and 6 SNPs, respectively). The distribution of genotyped SNPs (339) across 29 chromosomes of the bovine genome is presented in Figure 1.

### Association analysis

#### ***Single locus regression model (SLRM)***

Genotypic data were coded as 0, 1, 2 corresponding to the number of minor alleles using GenABEL. In this model, phenotypes were regressed on the number of copies of a minor allele (0, 1, or 2) for estimating the allele’s substitution effect using ASReml 3 software [20]. For feed efficiency traits, the univariate animal model was fitted as follows:

(2) $$\begin{array}{c} Y_{\mathrm{ijkl}}=µ+{\mathrm{Sex}}_{i}+{\mathrm{HY}}_{j}+\mathrm{TTYk}+\beta_{1}{\mathrm{SNP}}_{l}\\ \;+\beta_{2}{\mathrm{AET}}_{l}+\overset{l}{\underset{\mathrm{breed}=1}{\sum }}\beta_{l}\;\mathrm{breed}+\beta_{9}{\mathrm{HET}}_{l}\\ \;+a_{l}+e_{\mathrm{ijkl}} \end{array}$$

in which Y_ijkl_ is the trait measured in the l^th^ animal of the j^th^ herd-year of birth and the k^th^ treatment-trial-year group; μ is the overall mean for the trait; Sex_i_ is the fixed effect of the i^th^ sex of l^th^ animal; HY_j_ is the fixed effect of the j^th^ (17 level) herd-year of birth group; TTY_k_ is the fixed effect of the k^th^ (42 level) treatment trial-year of the test group; β_1_ is the regression coefficient of the linear regression on the number of copies of a minor allele; β_2_ is the regression coefficient of the linear regression on age at the end of the test period (AET) of the l^th^ animal; β_l_ is the regression coefficient of the linear regressions on proportion of AN, CH, LM, SM, PI, and GV breeds in the l^th^ animal; β_9_ is the regression coefficient of the linear regression on the percentage of heterozygosity of the l^th^ animal; a_l_ is the random additive genetic (polygenic) effect of the l^th^ animal; and e_ijklm_ is the residual random effect associated with the l^th^ animal. The TTY level that had less than three animals was excluded from the analysis. Phenotypes that were not within the mean ± 3 standard deviations for the respective trait were excluded from the analysis.

For carcass traits, the previous model (2) was modified to include the effect of the treatment trial-year-sex group instead of TTY and to include the fixed effect of the herd-year slaughter season instead of HY. Also, the effect of age at the end of the test period (day) was substituted by the age at slaughter (day).

The significance of associations was determined by an overall value of P < 0.05. To allow for multiple hypothesis-testing, chromosome wise false discovery rate (FDR) was used [21]. A threshold of 5 and 20% FDR were used for strong and suggestive associations, respectively.

#### ***Genotypic model***

This model was fitted only for feed efficiency traits to consider genetic effects other than the additive effect. The model included the same effects in the SLRM, except that the allele substitution effect was replaced with the genotype effect. This model was not fitted for carcass traits to reduce the volume of results as the trait of primary interest was feed efficiency for this study.

### Estimation of genetic variance explained by identified SNPs

The proportion of phenotypic variance in RFI explained by the full set of SNPs (339) that passed QC was estimated using the BayesC algorithm implemented in GenSel 3.13 software [22]. Also, the proportion of the genetic variance of RFI explained by the set of significant SNPs for at least one of the feed efficiency traits using SLRM and/or the genotypic model was estimated. Missing genotypes were inferred using fastPHASE [23]. Estimated breeding value (EBV) was determined with the SLRM without the regression on SNPs by ASReml. BayesC was then used to run the analysis with the two sets of SNPs (the full set [339 SNPs] and significant SNPs from the two models [98 SNPs]). Posterior residual and genetic variances were estimated after 41,000 iterations including 1,000 burn-in cycles. The proportion of genetic variance explained by the set of SNPs was estimated as the posterior genetic variance divided by phenotypic variance (posterior residual plus posterior genetic variance). In addition, the correlations between genomic breeding values predicted by estimated solutions and EBVs were estimated.

### Enrichment analysis

The significant (P < 0.05) SNPs (98) from the SLRM and genotype models for at least one feed efficiency trait from the association analysis were mapped to 74 genes. The list of the genes was submitted to DAVID 6.7 Beta software [24] for an *in silico* functional analysis. In DAVID, Gene ontology (GO) was used to identify functionally related genes. The genes were also mapped to biological pathways using web software in the Kyoto Encyclopedia of Genes and Genomes (KEGG) [25].

## Results and discussion

### Heritability estimates

Our goal in the current study was to identify informative or causal mutations for feed efficiency traits for use in Marker Assisted Selection (MAS). This would accelerate genetic improvement in beef cattle by improving the accuracy of selection and shortening intervals between generations [26]. Genetic Improvement of feed efficiency could subsequently minimize methane production [27] while optimizing beef production. In this study, a crossbred population was used to evaluate the relationship between potential genes identified from fine mapping and RFI. The descriptive statistics of feed efficiency, performance and carcass traits are given in Table 1. Using the single trait animal model in ASReml, the estimates of heritabilities are given in Table 2. Heritability estimates for feed efficiency traits are in the range reported in the literature. The estimated heritability for RFI (0.19) is within the reported range from 0.16 to 0.45 [28,29], whereas heritability (0.35) for ADG is in agreement with [30]. Estimated heritability for DMI (0.42) is within the reported range, from 0.31 to 0.44 [30,31]. FCR (0.25) is also within the reported range (0.17 to 0.37) [28,30] as is MMWT (0.48) from 0.36 to 0.69 [28,29]. The genetic variation and moderate heritabilities in feed efficiency traits indicate effective selection would be possible, where the trait is measured. However, the detected genetic variation also indicates that MAS could be effective where the genetic markers are closely linked to or is the causative mutation and that have repeatable effects across independent populations.

**Table 1:** Descriptive statistics (mean, SD, min and max) in feedlot beef cattle for feed efficiency, performance and carcass traits

**Trait**^**1**^	**No**^**2**^	**Mean**	**SD**	**Min**	**Max**
**Feed efficiency traits:**					
Average daily gain (ADG), kg d^-1^	726	1.70	0.385	0.71	3.30
Mid-test metabolic weight (MMWT), kg	726	92.4	11.70	53.3	128.1
Daily dry matter intake (DMI), kg d^-1^	726	9.81	1.76	4.18	15.54
Residual feed intake (RFI), kg d^-1^	726	-0.066	1.126	-3.70	3.35
Feed conversion ratio (FCR), kg gain kg^-1^ DM	726	6.09	1.87	3.11	16.76
**Carcass traits:**					
Hot carcass weight (HCW), kg	959	353.7	52.47	208	503
*Longissimus* muscle area (LMA), cm^2^	848	94.3	14.61	59.4	138.4
Lean meat within the rib section (LR), %	664	54.6	6.79	25.0	75.2
Lean yield grade (LY), %	846	60.1	2.78	51.0	65.0
Fat1 (F1), mm	850	13.4	5.62	1.0	30.0
Fat2 (F2), mm	850	15.7	6.49	1.0	36.0
Fat3 (F3), mm	847	9.6	3.66	1.0	22.0
Grade fat (GRF), mm	846	8.8	3.25	1.0	19.0
Proportion of intermuscular fat (IFR) within the rib section, %	687	10.09	3.22	1.2	20.5
Proportion of body cavity fat within the rib section (BFR), %	684	3.48	1.244	0.96	7.30
Proportion of subcutaneous fat from the rib section (SQFR), %	685	10.30	2.60	2.44	18.53
Marbling score^b^	851	4.90	0.734	3.0	6.0

^1^F1, subcutaneous fat depth between the 1^st^ and 2^nd^ quarter of the *longissimus*; F2, subcutaneous fat depth between 2^nd^ and 3^rd^ quarter of the *longissimus*; F3, subcutaneous fat depth between the 3^rd^ and 4^th^ quarter of the *longissimus*.

^2^No. = Number of animals’ phenotypes and genotypes for testing the association.

^b^Marbling was scored as ≤ 3.0 = devoid; 3.1 to 4.0 = traces; 4.1 to 5.9 = slight; 6.0 to 7.0 = small to moderate; and ≥ 7.0 = slightly abundant to abundant.

**Table 2:** **The heritability estimates (h**^**2**^**) ± standard error (SE) for growth and feed efficiency related traits estimated in crossbred beef cattle**

**Trait**^**1**^	**h**^**2**^ **± SE**
Average daily gain, kg d^-1^	0.35 ± 0.12
Mid-test metabolic weight, kg	0.48 ± 0.13
Daily dry matter intake, kg d^-1^	0.42 ± 0.17
Residual feed intake, kg d^-1^	0.19 ± 0.11
Feed conversion ratio, kg gainkg^-1^ DM	0.25 ± 0.13
Hot carcass weight (HCW), kg	0.29 ± 0.10
*Longissimus* muscle area (LMA), cm^2^	0.50 ± 0.11
Lean meat within the rib section (LR), %	0.48 ± 0.13
Lean yield grade (LY), %	0.31 ± 0.10
Fat1 (F1), mm	0.10 ± 0.08
Fat2 (F2), mm	0.24 ± 0.10
Fat3 (F3), mm	0.22 ± 0.10
Grade fat (GRF), mm	0.24 ± 0.10
Proportion of intermuscular fat (IFR) within the rib section, %	0.54 ± 0.14
Proportion of body cavity fat within the rib section (BFR), %	0.23 ± 0.12
Proportion of subcutaneous fat from the rib section (SQFR), %	0.20 ± 0.12
Marbling score,^b^	0.41 ± 0.10

^1^F1, subcutaneous fat depth between the 1^st^ and 2^nd^ quarter of the *longissimus*; F2, subcutaneous fat depth between 2^nd^ and 3^rd^ quarter of the *longissimus*; F3, subcutaneous fat depth between the 3^rd^ and 4^th^ quarter of the *longissimus*.

^b^Marbling was scored as ≤ 3.0 = devoid; 3.1 to 4.0 = traces; 4.1 to 5.9 = slight; 6.0 to 7.0 = small to moderate; and ≥ 7.0 = slightly abundant to abundant.

### Association analysis

In the current study, SNP effects were estimated using an allele substitution effect model or the genotypic model. To avoid population stratification effects from influencing the estimated SNP effect, the phenotypes were adjusted for breed proportion, and the polygenic effect was fitted using the animal model to account for possible family effects [32]. The population used in the GWAS using the Illumina BovineSNP50 was different animals to those used in the current study. However, the two populations are not independent as the animals in the current study were born subsequent to the animals used in the GWAS population from the same primary herds. The average relatedness among individuals between the two populations was estimated to be low at 0.0005 on average using the numerator relationship matrix calculated using CFC [33]. There was zero pedigree-based inbreeding among the animals used in the current study. There was no separate dataset for feed efficiency traits on the same 339 SNP chip that could be used for validation. Thus, these significant associations require validation in other independent populations.

Results indicated 15 SNPs were significantly (at less 5% FDR) associated with at least one feed efficiency trait phenotype using the allele substitution effect model (Table 3). These findings reveal several candidate genes that provide highly significant evidence of association with RFI (Table 3). These promising candidate genes are located on *Bos taurus* autosomes (BTA) 8, 15, 16, 18, 19, 21, and 28. The strongest evidence of association with RFI and DMI was in SNP (8: 10674426) in the three prime untranslated region (3′ UTR) of gene elongation protein 3 homolog (*ELP3*). Gene *ELP3* modulates transcription by working as a catalytic histone acetyltransferase subunit of the RNA polymerase II elongator complex involved in transcriptional elongation [34,35]. In Drosophila, reduction in ELP3 expression during the development of the nervous system increases activity and decreases sleep [36] and the growth of adult flies (or could be lethal for the pupa) [37].

**Table 3:** Significant and suggestive SNP based on false discovery rate (FDR) q threshold of 0.05 and 0.2 for feed efficiency traits using single locus regression model (SLRM) on 339 SNPs

**Trait**^**1**^	**Gene ID**^**2**^	**BTA**^**3**^	**Ref. SNP**^**4**^	**Pos. ( bp)**^**5**^	**MAF**^**6**^	**Alleles**^**7**^	**n**^**8**^	**Estimate ± SE**^**9**^	***P-value***
ADG	523789	3	rs42417924	75523597	0.102	C/G	726	-0.062 ± 0.02	0.0104&
DMI	616055	5	ss914082855	119551668	0.37	A/G	726	-0.234 ± 0.08	0.0025^*^
DMI	616055	5	ss914082856	119557146	0.37	T/C	720	-0.237 ± 0.08	0.0023^*^
MMWT	616908	6	rs41574929	36099801	0.388	T/G	716	0.992 ± 0.40	0.0132&
ADG	616908	6	rs41574929	36099801	0.388	T/G	716	0.049 ± 0.01	0.0009^*^
MMWT	540329	6	ss914082878	37288379	0.152	T/C	726	1.342 ± 0.54	0.0128&
MMWT	536203	6	ss914082880	37386084	0.16	A/G	725	1.293 ± 0.53	0.0148&
MMWT	530393	6	rs43702346	37439120	0.272	T/G	723	1.421 ± 0.43	0.0009^*^
DMI	784720	8	ss914082889	10674426	0.041	A/G	726	0.620 ± 0.18	0.0006^*^
RFI	784720	8	ss914082889	10674426	0.041	A/G	726	0.483 ± 0.15	0.0017^*^
MMWT	282689	10	ss914082689	50256553	0.197	T/C	725	-1.210 ± 0.48	0.01&
MMWT	282689	10	ss914082690	50259055	0.046	A/G	726	2.184 ± 0.89	0.015&
MMWT	614507	10	ss914082694	79315960	0.364	T/C	718	0.933 ± 0.39	0.0176&
RFI	533166	15	rs41755948	30710940	0.207	T/C	726	-0.201 ± 0.07	0.007&
RFI	533166	15	ss914082737	30717928	0.207	T/C	726	-0.201 ± 0.07	0.007&
RFI	521326	16	rs41821600	64875340	0.037	A/T	726	0.496 ± 0.17	0.0033^*^
RFI	521326	16	rs41820824	64950387	0.012	A/G	726	0.785 ± 0.29	0.0064^*^
RFI	508025	18	ss914082760	17150858	0.365	T/C	723	0.191 ± 0.06	0.0028^*^
FCR	282411	19	rs41914675	37278418	0.072	A/G	726	0.278 ± 0.10	0.004&
RFI	282411	19	rs41914675	37278418	0.072	A/G	726	0.342 ± 0.12	0.004&
DMI	282411	19	rs41914675	37278418	0.072	A/G	726	0.462 ± 0.14	0.0008^*^
RFI	524684	21	rs43020736	29054823	0.371	T/C	726	-0.162 ± 0.07	0.016&
DMI	524684	21	rs43020736	29054823	0.371	T/C	726	-0.248 ± 0.08	0.0018^*^
RFI	524684	21	rs43020769	29060759	0.478	A/G	726	0.172 ± 0.07	0.009&
MMWT	532512	25	ss914082815	36278405	0.11	T/C	719	1.555 ± 0.63	0.014&
MMWT	532512	25	ss914082816	36279504	0.02	T/C	726	3.099 ± 1.31	0.018&
MMWT	515895	27	ss914082827	39798548	0.08	A/G	726	-2.298 ± 0.71	0.0013^*^
DMI	508697	28	ss914082834	7727734	0.426	A/G	726	-0.211 ± 0.08	0.0067^*^
RFI	508697	28	ss914082834	7727734	0.426	A/G	726	-0.183 ± 0.07	0.005^*^
RFI	780878	28	ss914082829	13580673	0.187	T/A	726	-0.208 ± 0.08	0.009^*^

^1^average daily gain (ADG), kg d^-1^, average daily dry matter intake (DMI), kg d^-1^, mid-point metabolic weight (MMWT), kg^75^, feed efficiency conversion ratio (FCR), kg gainkg-^1^ DM and residual feed intake (RFI) kg d^-1^.

^*^is a significant SNP after adjusting for chromosome-wise 5% false discovery rate.

& is a suggestive SNP after adjusting for chromosome-wise 20% false discovery rate.

Gene ID^2^ = Entrez gene identifier.

BTA^3^ = *Bos taurus* autosome.

Ref. SNP^4^ = (rs#) is a reference SNP ID number and (ss#) ID is the National Center for Biotechnology Information (NCBI) assay ID number assigned by NCBI to submitted SNPs for discovered SNPs using RNA-Seq.

Pos. (bp)^5^ = the SNP’s position in a base pair.

MAF^6^ = minor allele frequency.

Alleles^7^ = first allele/second allele, the second allele is the minor allele which the phenotypes regressed on its number (0, 1, and 2).

n^8^ = Number of animals’ phenotypes and genotypes for testing the association.

Estimate ± SE^9^ = allele substitution effect ± standard error.

In the current study on BTA 16, the splice site intronic mutation (rs41820824) and the missense mutation (rs41821600) within gene hemicentin 1(*HMCN1*) were associated with RFI, where the substitution with the minor allele was associated with increased RFI and decreased F1. In addition, the minor allele of SNP rs41824268, within gene *HMCN1,* was associated with decreasing HCW, whereas in SNP rs41820800, it was associated with decreasing F2. Gene HMCN1 is known to be involved in age-related, macular degeneration [38], and polymorphisms within gene *HMCN1* were associated with diabetes in man [39].

Synonymous coding SNP (18: 17150858), within the gene encoding zinc finger protein 423 (*ZNF423*), was associated with RFI, DMI and MMWT and located near a reported QTL (ID: 4449) for DMI [6]. In addition, the minor allele of SNP (18: 17152044) was associated with decreasing GRF, F3, and F2, and increased LY, and was located near a reported QTL (ID: 11062) for LMA and body weight (ID: 11061) [40]. Gene *ZNF423* is a transcription factor involved in metal ion-binding. Down regulation of *ZNF423* expression increases cell growth and retards differentiation as a consequence of its important role with the Vitamin A metabolite, retinoic acid [41].

On BTA 6, the SNP (6: 37288379) at 3′ UTR, within gene protein phosphatase, Mg^2+/^Mn^2+^ dependent, 1 K (*PPM1K*), was associated with increased MMWT and HCW and decreased RFI, FCR, marbling, and IFR and was located near a reported QTL (ID: 10761) for fat thickness at the 12^th^ rib and a QTL (ID: 10758) for marbling score (EBV) [40] and a QTL (ID: 1753) for milk fat percentage [42]. Gene *PPM1K* is involved in the phosphorus metabolic process or in amino acid dephosphorylation. In addition, *PPM1K* plays a key role in cellular survival and development by regulating mitochondrial permeability transition pore function [43]. However, different genes are involved in mitochondrial adenosine triphosphate (ATP) synthesis efficiency and associated with differences in RFI [44-46], therefore, the effect of gene *PPM1K* on mitochondrial ATP synthesis is not clear [43].

For ADG, the most significant (at less than 5% FDR; P = 0.0009) SNP (rs41574929) was located on BTA 6, at 5′ UTR, within gene family with sequence similarity 190, member (A FAM190A; ID: 616908) (Table 3). The SNP rs41574929 was also associated ssignificantly at less than 5% FDR with HCW (P = 0.006). This result is in agreement with the function described for FAM190A where it is a necessary regulator for normal mitosis [47]. A deletion mutation in FAM190A causes a cell division defect [47].

Allele substitution effect estimates of SNPs influencing (P ≤ 0.05) growth and efficiency traits, but which did not pass chromosome wise false discovery rate (FDR) threshold q = 0.2 were listed in Additional file 2. Also, all the SNPs associated at *P*-value < 0.05 using the genotypic model for growth and feed efficiency traits were listed in Additional file 3.

The association analysis using SLRM indicated that 59 SNPs were strongly (Table 4) or suggestively (Additional file 4) associated at 5% or 20% FDR test, respectively, for at least one carcass trait phenotype. Results indicated that the majority of the strong or suggestive associations were for intermuscular fat % (IFR) (14 indications). Thirteen SNPs were strongly associated with marbling, whereas 12 SNPs were associated with longissimus muscle area (LMA), and as follows HCW (9), F3 (8), GRF (8), F2 (6), LY (6), body cavity fat within the rib section (BFR) (5), % lean meat within the rib section (LR) (4), % subcutaneous fat within the rib section (SQFR) (4), and F1 (2).

**Table 4:** Significant SNP based on false discovery rate (FDR) q threshold of 0.05 for beef carcass traits using single locus regression model

**Trait**^**1**^	**Gene ID**^**2**^	**BTA**^**3**^	**Ref. SNP**^**4**^	**BPPos**^**5**^	**MAF**^**6**^	**n**^**7**^	**Estimate ± SE**^**8**^	***P-*** value^*^
LR	539020	1	rs43246339	81372644	0.165	664	1.151 ± 0.266	0.00002
IFR	539020	1	rs43246339	81372644	0.167	687	-0.622 ± 0.172	0.0003
IFR	614882	2	rs43287969	1280728	0.329	648	0.404 ± 0.143	0.005
F3	532545	2	rs43307594	43392336	0.379	847	0.473 ± 0.15	0.0016
IFR	538378	2	rs42315485	58475918	0.035	687	-0.968 ± 0.355	0.0065
Marbling	522946	3	ss914082840	2555332	0.445	851	-0.097 ± 0.028	0.00066
Marbling	522946	3	ss914082841	2557106	0.379	837	-0.092 ± 0.031	0.003
LMA	532836	4	rs41599809	96565402	0.107	848	3.08 ± 0.975	0.0016
LMA	532836	4	ss914082853	96570062	0.37	848	-1.807 ± 0.617	0.0035
Marbling	538086	5	ss914082862	50301829	0.414	851	-0.091 ± 0.03	0.0025
IFR	503621	6	ss914082876	32016672	0.131	687	0.625 ± 0.191	0.001
HCW	616908	6	rs41574929	36099801	0.397	949	5.277 ± 1.916	0.006
Marbling	540329	6	ss914082878	37288379	0.166	851	-0.117 ± 0.038	0.002
IFR	540329	6	ss914082878	37288379	0.14	687	-0.535 ± 0.178	0.0028
HCW	540329	6	ss914082878	37288379	0.162	959	7.16 ± 2.468	0.0038
Marbling	536203	6	ss914082880	37386084	0.174	850	-0.121 ± 0.037	0.001
IFR	536203	6	ss914082880	37386084	0.152	685	-0.511 ± 0.172	0.003
HCW	530393	6	rs29010894	37433382	0.124	958	-7.542 ± 2.706	0.005
IFR	530393	6	rs43702346	37439120	0.276	684	-0.393 ± 0.14	0.005
SQF	616568	7	ss914082884	10135670	0.129	928	-0.022 ± 0.008	0.004
GRF	616568	7	ss914082884	10135670	0.13	819	-0.572 ± 0.206	0.0056
Fat3	541122	9	rs43013785	33837458	0.487	841	0.431 ± 0.148	0.004
IFR	529759	11	ss914082698	80982741	0.066	687	0.928 ± 0.28	0.00097
Marbling	537649	12	ss914082709	13011713	0.294	851	0.125 ± 0.033	0.0002
F3	537649	12	ss914082709	13011713	0.293	847	0.443 ± 0.171	0.0097
IFR	535653	12	rs43694364	15748029	0.483	687	0.379 ± 0.131	0.0039
GRF	509602	12	ss914082715	76885563	0.411	845	0.409 ± 0.137	0.0029
LR	509602	12	ss914082715	76885563	0.425	663	-0.584 ± 0.201	0.0037
F3	509602	12	ss914082715	76885563	0.411	846	0.431 ± 0.15	0.004
IFR	509602	12	ss914082716	76889667	0.413	686	-0.503 ± 0.135	0.0002
LYR	509602	12	ss914082716	76889667	0.413	663	0.748 ± 0.208	0.0003
SQFR	509602	12	ss914082716	76889667	0.414	684	-0.355 ± 0.119	0.003
F3	509602	12	ss914082716	76889667	0.417	846	-0.439 ± 0.153	0.004
GRF	509602	12	ss914082716	76889667	0.418	845	-0.381 ± 0.14	0.0065
F3	512287	15	ss914082741	4101726	0.099	843	0.865 ± 0.254	0.00068
Fat1	521326	16	rs41821600	64875340	0.038	849	-2.099 ± 0.627	0.00085
Marbling	540672	20	rs43006895	54577104	0.479	851	0.1 ± 0.029	0.0006
F2	540672	20	rs43006895	54577104	0.478	850	0.904 ± 0.287	0.0017
BFR	534312	21	rs41980260	33909131	0.282	683	-0.162 ± 0.057	0.0049
BFR	534312	21	rs41980261	33909583	0.282	684	-0.151 ± 0.057	0.008
LMA	512725	23	ss914082809	32192762	0.488	848	1.609 ± 0.611	0.0086
LMA	512725	23	ss914082812	32207295	0.283	848	1.84 ± 0.703	0.009
LMA	504741	24	rs42047790	36412358	0.445	848	2.087 ± 0.625	0.0009
LMA	540050	26	rs42106947	37495213	0.488	848	-1.766 ± 0.611	0.004
BFR	518905	27	ss914082824	39712547	0.015	684	0.714 ± 0.231	0.002
IFR	515895	27	ss914082827	39798548	0.076	687	0.654 ± 0.237	0.006
SQFR	515895	27	ss914082827	39798548	0.077	685	0.569 ± 0.208	0.006

^1^F1, subcutaneous fat depth between the 1^st^ and 2^nd^ quarter of the *longissimus*; F2, subcutaneous fat depth between 2^nd^ and 3^rd^ quarter of the *longissimus*; F3, subcutaneous fat depth between the 3^rd^ and 4^th^ quarter of the *longissimus*; Marbling was scored as ≤ 3.0 = devoid; 3.1 to 4.0 = traces; 4.1 to 5.9 = slight; 6.0 to 7.0 = small to moderate; and ≥ 7.0 = slightly abundant to abundant; HCW = Hot carcass weight (kg); LMA = longissimus dorsi muscle area (cm2); LR = lean meat within the rib section (%); LY = Lean yield grade (%); GRF = Grade fat (mm); IFR = Intermuscular fat (%); BFR = Body cavity fat within the rib section (%); SQFR = Proportion of subcutaneous fat from the rib section (%); ^*^is a significant SNP after adjusting for chromosome-wise 5% false discovery rate.

Gene ID^2^ = Entrez gene identifier; BTA^3^ = Bos taurus autosome; Ref. SNP^4^ = (rs#) is a reference SNP ID number and (ss#) ID is the National Center for Biotechnology Information (NCBI) assay ID number assigned by NCBI to submitted SNPs for discovered SNPs using RNA-Seq.

BPPos^5^ = the SNP’s position in a base pair; MAF^6^ = minor allele frequency; n^7^ = Number of animals’ phenotypes and genotypes for testing the association; Estimate ± SE^8^ = allele substitution effect ± standard error, the minor allele which the phenotypes regressed on its number (0, 1, and 2).

Significant effects (at less 5% FDR) were found in 27 genes where gene ERCC5 (ID: 509602) had the highest proportion of the effects, revealing 8 of the significant associations with carcass traits (Table 4). The newly discovered SNP on BTA 12 (76889667 bp), within gene ERCC5 (ID: 509602), provided evidence of association with 5 of the studied carcass traits where the substitution of the minor allele was associated with increases of LYR and decreases in marbling, F3, GRF, IFR and SQFR (Table 4). Another newly discovered SNP in gene ERCC5 (76885563 bp) was strongly associated with three carcass traits where the substitution of the minor allele was associated with increases in F3, and GRF and decreases in LR (Table 4). Gene ERCC5 is involved in response to abiotic stimulus and negative regulation of programmed cell death and nucleotide excision repair pathway. In mice selected for high muscle mass, ERCC5 was located in QTL for lean mass [48].

SNP on BTA 27 (39712547 bp), within gene solute carrier family 20 (phosphate transporter), member 2 (*SLC20A2*; ID: 518905), was associated with one carcass trait where the substitution of the minor allele was associated with an increase in BFR (Table 4). The *SLC20A2* is involved in ion and cation transport. In human, mutations within *SLC20A2* are associated with idiopathic basal ganglia calcification [49].

SNP rs43702346 on BTA 6, within gene polycystic kidney disease 2 (*PKD2*; ID: 530393), was significantly associated with two carcass traits where substitution with the minor allele was associated with a decrease in HCW and IFR (Table 4). The *PKD2* gene is involved in negative regulation of G1/S transition of mitotic cell cycle process. Gene *PKD2* is near an identified QTL for bone percentage, fat percentage, meat percentage, meat-to-bone ratio, moisture content and subcutaneous fat [50]. In human, polymorphisms within *PKD2* may take part in the development of gout [51].

### The *in silico* functional analysis

In the current study, the 74 genes containing significant (P < 0.05) SNPs were submitted to DAVID for enrichment analysis. In total 39 genes out of the 74 genes were enriched in 35 biological process terms (Table 5). Ion transport and cation transport mechanisms contained the highest number of genes associated with feed efficiency traits. In addition, some genes affecting feed efficiency traits in the current study were involved in proteolysis, protein complex biogenesis, and protein amino acid glycosylation. The ion transport mechanism in conjunction with protein turnover and metabolism account for 37% of the variation in RFI [52].

**Table 5:** **Enriched biological processes for 39 genes holding significant SNPs ( *P *-value < 0.05) for feed efficiency traits**

**Biological process**	**No**	**P value**^**‡**^	**Genes**
Ion transport	8	0.006	618639, 518905, 281701, 530393, 540113, 510792, 282411, 614299
Cation transport	7	0.004	618639, 518905, 530393, 540113, 510792, 282411, 614299
Phosphorus metabolic process	7	0.057	504429, 533815, 540329, 540113, 100048947, 281848, 512125
Phosphorylation	6	0.072	504429, 533815, 540113, 100048947, 281848, 512125
Metal ion transport	5	0.034	618639, 518905, 530393, 282411, 614299
Regulation of transcription	5	0.762	517336, 509259, 529124, 540474, 784720
Protein amino acid phosphorylation	5	0.126	504429, 533815, 100048947, 281848, 512125
Monovalent inorganic cation transport	4	0.060	618639, 518905, 540113, 614299
Regulation of transcription, DNA-dependent	4	0.722	517336, 529124, 540474, 784720
Transmembrane transport	4	0.236	512725, 281701, 540113, 282411
Proteolysis	3	0.756	617222, 524684, 534774
Intracellular signalling cascade	3	0.643	530393, 614507, 281848
Regulation of transcription from RNA polymerase II promoter	3	0.290	517336, 540474, 784720
Transcription	3	0.636	509259, 529124, 784720
RNA processing	3	0.320	100048947, 512925, 281712
Potassium ion transport	2	0.359	618639, 614299
Nucleobase, nucleoside, nucleotide and nucleic acid biosynthetic process	2	0.497	540113, 510792
Calcium ion transport	2	0.279	530393, 282411
Regulation of homeostatic process	2	0.152	530393, 282411
Response to abiotic stimulus	2	0.448	509602, 530393
Negative regulation of programmed cell death	2	0.476	509602, 282032
Protein complex biogenesis	2	0.545	509259, 281848
Determination of symmetry	2	0.067	497208, 530393
Microtubule-based process	2	0.441	497208, 512287
Blood vessel morphogenesis	2	0.351	282689, 282032
Protein transport	2	0.833	282044, 614507
Protein amino acid autophosphorylation	2	0.130	281848, 512125
Neurological system process	2	0.679	281701, 538198
Oxidation reduction	2	0.899	280951, 532512
mNRA metabolic process	2	0.495	100048947, 281712
Cell-cell adhesion	1	1.000	540672
Protein amino acid glycosylation	1	1.000	532545
Muscle cell development	1	1.000	529759
Amino acid transport	1	1.000	511955

^‡^P value of the enriched biological process for genes’ list having significant SNP.

In ruminants protein synthesis accounts for 23% of total energy use in the whole body[53] and protein turnover accounts for 42% of total gastrointestinal tract energy use [54]. In the current study, some genes were involved in phosphorus metabolic processes, phosphorylation, and amino acid phosphorylation. Protein metabolism can be controlled by changing the phosphorylation status [55]. Genes involved in phosphorus metabolic processes and phosphorylation mechanisms regulate the metabolism of energy [56]. In the current study, regulation of transcription mechanisms contributed to variation in feed efficiency traits. The connection between a functional mutation in a specific transcription factor can increase or decrease expression of genes involved in glucose, amino acid, lipid, and cholesterol metabolism [57]. Other studies have demonstrated that genes that up-regulate in response to nutritional restriction are involved in transcription control [58].

The *in silico* functional study of genes having significant SNPs revealed potential pathways likely to contribute to variation in feed efficiency traits (Table 6). Mitogen-activated protein kinases (MAPK) signaling pathway included three of the identified genes (*RASA1*, *CACNA1G* and *STK3*). In a study of the differences in global gene expression between high and low RFI animals, the majority of up-regulated genes in low RFI animals were stimulated by MAPKs [59], where the MAPKs were involved in signal transduction pathways to activate different cellular processes, such as cell division, differentiation, and cell death as a response to hormones and stress [60]. The TYR gene is involved in Riboflavin metabolism, melanogenesis, tyrosine metabolism, and catecholamine biosynthesis, and the minor allele of SNP rs42402428, within gene TYR (ID: 280951) was associated with decreasing FCR. Polymorphisms in gene TYR have been associated with changing the coat colour of Braunvieh cattle [38]. Gene *GALNT13,* affecting ADG, MMWT, DMI, F2, GRF, HCW, LMA, LY, and F3, is involved in mucin type O-Glycan biosynthesis. Gene *ATP6V1E2* (ID: 540113), which affects DMI and MMWT, plays an important role in various pathways and biological mechanisms. Gene *ATP6V1E2* is near an identified QTL for *mycobacterium avium spp. Paratuberculosis* resistance in Holstein cattle [61]. Gene *GTF2F2* (ID: 509259) affected RFI and is involved in basal transcription factors pathways, which regulate glucose, amino acids and protein, lipid metabolism and many other important metabolic processes. Changes in the function of *GTF2F2* would be associated with feed efficiency or metabolic diseases [57]. The minor allele of a newly discovered SNP (6: 37386084), within gene *ABCG2* (ID: 536203), was associated with decreasing IFR and marbling. The *in silico* functional analysis showed that gene *ABCG2* is involved in ATP-binding cassette (ABC) transporters and bile secretion pathways. The results of gene *ABCG2* in the current study agree with reported gene *ABCG2* as QTL for increasing milk yield and decreasing milk fat and protein [62-64]. The analysis also indicated that insulin-like growth factor 1 receptor gene *IGF1R* (ID: 281848) affecting ADG and marbling is involved in seven different pathways. Nonetheless, there was no association between production traits and the genotypes of IGF-IR/TaqI polymorphism [65-67]. This might be because a small number of animals was used to test the association in those analyses. Functional analysis allows a better understanding of the underlying mechanisms contributing to the genetic variation in feed efficiency, and it sheds light on potential pathways to target in future investigations.

**Table 6:** The pathways for 14 genes containing significant SNPs for one feed efficiency trait

**Pathway**	**Genes**
bta04010: MAPK signalling pathway	282032 (*RASA1*), 282411 (*CACNA1G*), 533815 (*STK3*)
bta01100: Metabolic pathways	280951 (*TYR*), 532545 (*GALNT13*), 540113 (*ATP6V1E2*)
bta04930: Type II diabetes mellitus	282411 (*CACNA1G*), 538996 (*ABCC8*)
bta04145: Phagosome	512287 (*LOC512287*), 540113 (*ATP6V1E2*)
bta02010: ABC transporters	536203 (*ABCG2*), 538996 (*ABCC8*)
bta04976: Bile secretion	536203 (ABCG2)
bta03013: RNA transport	616055 (CHADL)
bta03022: Basal transcription factors	509259 (GTF2F2)
bta04962: Vasopressin-regulated water reabsorption	512287 (LOC512287)
bta05132: Salmonella infection	512287 (LOC512287)
bta04514: Cell adhesion molecules (CAMs)	529759 (SDC1)
bta04512: ECM-receptor interaction	529759 (SDC1)
bta05144: Malaria	529759 (SDC1)
bta04966: Collecting duct acid secretion	540113 (ATP6V1E2)
bta04721: Synaptic vesicle cycle	540113 (ATP6V1E2)
bta00190: Oxidative phosphorylation	540113 (ATP6V1E2)
bta05323: Rheumatoid arthritis	540113 (ATP6V1E2)
bta03420: Nucleotide excision repair	509602 (ERCC5)
bta04510: Focal adhesion	281848 (IGF1R)
bta04114: Oocyte meiosis, bta05214: Glioma	281848 (IGF1R)
bta05218: Melanoma, bta05200: Pathways in cancer	281848 (IGF1R)
bta04914: Progesterone-mediated oocyte maturation	281848 (IGF1R)
bta04520: Adherens junction	281848 (IGF1R)
bta04730: Long-term depression,	281848 (IGF1R)
bta04144: Endocytosis	281848 (IGF1R)
bta00740: Riboflavin metabolism	280951 (TYR)
bta04916: Melanogenesis	280951 (TYR)
bta00350: Tyrosine metabolism	280951 (TYR)
bta03015: mRNA surveillance pathway	281712 (CPSF3)
bta03008: Ribosome biogenesis in eukaryotes	508697 (HEATR1)
bta05010: Alzheimer’s disease	534774 (BACE2)
bta04360: Axon guidance	282032 (RASA1)
bta04740: Olfactory transduction	281701 (CNGA3)
bta00512: Mucin type O-Glycan biosynthesis	532545 (GALNT13)
bta05164: Influenza A	100048947 (RNASEL)
bta05160: Hepatitis C	100048947 RNASEL
bta04020: Calcium signalling pathway	282411 (CACNA1G)

### Genetic variation in RFI explained by candidate genes

The accuracy of a DNA panel to predict a trait like feed efficiency depends on the amount of genetic variation explained. The 98 SNP set associated (P < 0.05) with at least one feed efficiency trait included SNPs that did not pass the FDR threshold, although they significantly contributed towards building the prediction equation in GWAS. The 98 SNP set explained 26% of the genetic variance in RFI whereas the proportion explained by the set of 339 SNPs was 29.6%. The correlation between EBVs of RFI using ASReml and GEBV were 0.52 and 0.66 from the 98 and 399 SNP sets, respectively. Based on the proportion of the genetic variance explained by the 98 SNPs (26%), the corresponding Beef Improvement Federation (BIF) accuracy is 0.127. Nonetheless, the estimated genetic variance by the 98 SNPs might be overestimated as the additive polygenic animal effect was not included in the model. To improve the accuracy of the SNP panel developed from a crossbred population, a large number of phenotypes is required (~2000 animals) [68]. This might partially explain the relatively low estimated accuracy in the current study. In addition, large numbers of identified genes (83 out of 180) from fine mapping RFI were genotyped for only one SNP, and that decreases the probability of detecting the functional mutations. Nonetheless, combining validated SNPs from further fine mapping and the identified 98 SNPs may help develop a DNA test panel for commercial use.

## Conclusion

This study reported SNPs that are significantly associated with RFI, performance, and carcass traits. We postulated that the identified significant SNPs, genes, biological mechanisms and pathways could be the direct cause of the variations in feed efficiency traits and carcass traits. The ability of the significant SNP to predict the genetic merit of feed efficiency and carcass traits should be measured in another population.

## Abbreviations

SNP: Single nucleotide polymorphism; QTL: Quantitative trait loci; FDR: False discovery rate; BTA: Bos taurus autosome; RFI: Residual feed intake; ADG: Average daily gain; DMI: Average daily dry matter intake, MMWT, Mid-point metabolic weight; FCR: Feed efficiency conversion ratio (kg gain kg^-1^ DM); F1: Subcutaneous fat depth between the 1st and 2nd quarter of the longissimus; F2: Subcutaneous fat depth between 2nd and 3rd quarter of the longissimus; F3: Subcutaneous fat depth between the 3rd and 4th quarter of the longissimus; HCW: Hot carcass weight (kg); LMA: Longissimus dorsi muscle area (cm2); LR: Lean meat within the rib section (%); LY: Lean yield grade (%); GRF: Grade fat (mm); IFR: Intermuscular fat (%); BFR: Body cavity fat within the rib section (%); SQFR: Proportion of subcutaneous fat from the rib section (%); EBRC: Elora Beef Research Center; KAP: the Agriculture and Agri-Food Canada Kapuskasing Research Centre; NLARS: New Liskeard Agriculture Research Station.

## Competing interests

The authors declare that they have no competing interests.

## Authors’ contributions

MKA contributed in designing the study, preparing the phenotypes and genotypes, performing the statistical and enrichment analysis, and drafting the manuscript. RV performed the data editing and statistical analysis. GV and IBM provided help in collection of data, analysis and manuscript editing. EJS and KCS participated in designing the study, collection of data and manuscript editing. PS, SM, GP participated in designing the study, preparing the genotypes, and editing the manuscript. SPM helped in design, data collection, analysis, and draft the manuscript. All authors’ read and approved the final manuscript.

## Supplementary Material

Additional file 1 The list of 339 genotyped single nucleotide polymorphisms (SNPs) and their related information. Click here for file

Additional file 2 Allele substitution effect estimates of SNPs influencing (P≤ 0.05) growth and efficiency traits, and not passed chromosome wise false discovery rate (FDR) threshold q=0.2. Click here for file

Additional file 3 Single nucleotide polymorphisms associated at P-value < 0.05 using the genotypic model for growth and feed efficiency traits. Click here for file

Additional file 4 Suggestive SNP based on false discovery rate (FDR) q threshold of 0.2 for beef carcass traits using single locus regression model (SLRM). Click here for file
